# Machine Vision to Provide Quantitative Analysis of Meltpool Stability for a Coaxial Wire Directed Energy Deposition Process

**DOI:** 10.3390/ma17215311

**Published:** 2024-10-31

**Authors:** Braden McLain, Remy Mathenia, Todd Sparks, Frank Liou

**Affiliations:** 1 Department of Mechanical and Aerospace Engineering, Missouri University of Science and Technology, Rolla, MO 65409, USA; btmywv@mst.edu (B.M.); remymathenia@mst.edu (R.M.); 2 Product Innovation and Engineering, St. James, MO 65559, USA; toddesparks@mopine.com

**Keywords:** laser-wire deposition, machine vision, additive manufacturing, stability, stubbing, dripping

## Abstract

Wire-based additive manufacturing (AM) is at the forefront of complex metal fabrication because of its scalability for large components, potential for high deposition rates, and ease of use. A common goal of wire directed energy deposition (DED) is preserving a stable process throughout deposition. If too little energy is put into the deposition, the wire will stub into the substrate and begin oscillating, creating turbulence within the meltpool. If too much energy exists, the wire will overheat, causing surface tension to take over and create liquid drips as opposed to a solid bead. This paper proposes a computer vision technique to work as both a state detection and event detection system for wire stability. The model utilizes intensity variations along with frame-to-frame difference calculations to determine process stability. Because the proposed model does not rely on machine learning techniques, it is possible for an individual to interpret and adjust as they see fit. The first part of this paper describes creation and implementation of the model. The model’s capability was then evaluated using a 1D laser power experiment, which generated a wide range of stability states across varying powers. The model’s accuracy was evaluated through 3D geometry data gathered from the experimentally deposited beads. The model proved to be both capable and accurate and has potential to be used as a real-time control system with future work.

## 1. Introduction

Additive manufacturing (AM) has garnered significant attention in the manufacturing research community because of its promises of improved design flexibility, reduced material waste, and rapid prototyping applications. The last 30 years of research have begun to flourish into an expanse of commercial activity including the automotive, aerospace, biomedical, and energy industries [1]. The automotive and aerospace industries are particularly interested in a specific AM technique known as directed energy deposition (DED). DED is attractive because of its high deposition rates and resultant ability to build large-scale parts [2]. DED feeds a stream of input material into a concentrated energy source in order to melt and shape incoming feedstock into a desired geometry. The two primary DED feedstock options are powder and wire. Wire-based DED has numerous benefits over powder-based DED systems including higher deposition rates, lower material costs, greater material availability, and lower propensity for porosity inclusions [3]. Defects such as gas porosity inclusions and lack of fusion are essential factors when qualifying critical components for use. Learning ways to reduce these defects through the use of process parameter adjustments and feedback control is a key active research interest.

**Figure 1:**
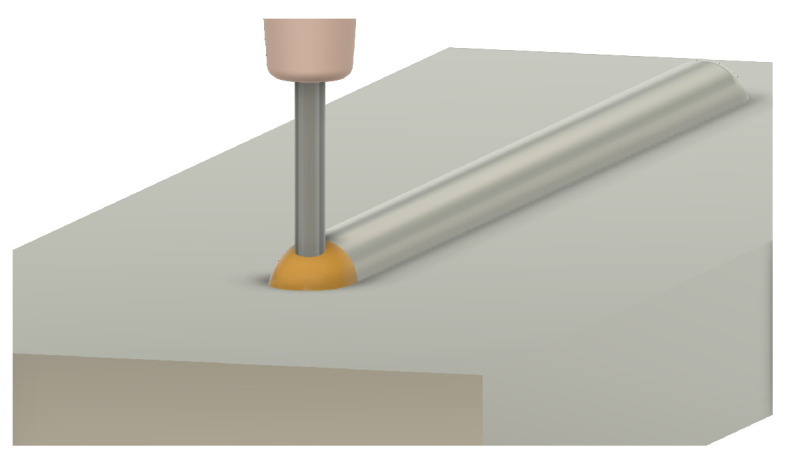
Three-dimensional rendering of an ideal stable deposition. The bead profile is uniform throughout its length and the wire is centered in the meltpool.

**Figure 2:**
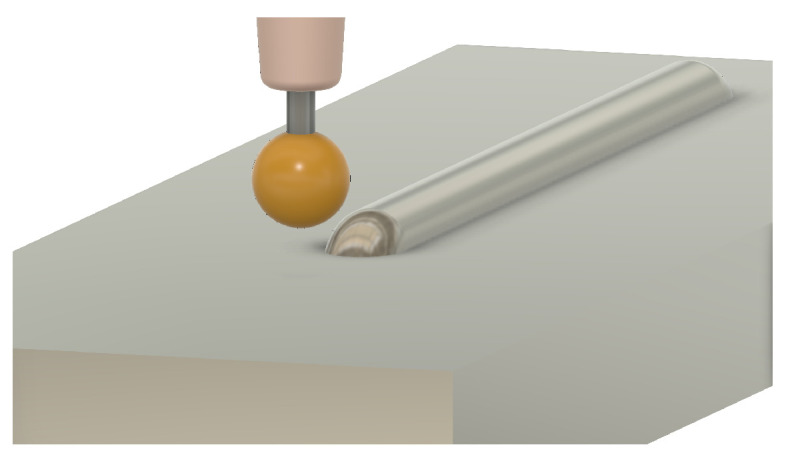
Three-dimensional rendering of a deposition that transitioned from stable to dripping. The wire has lifted off of the substrate, creating a discontinuity between the deposited bead and incoming material.

**Figure 3:**
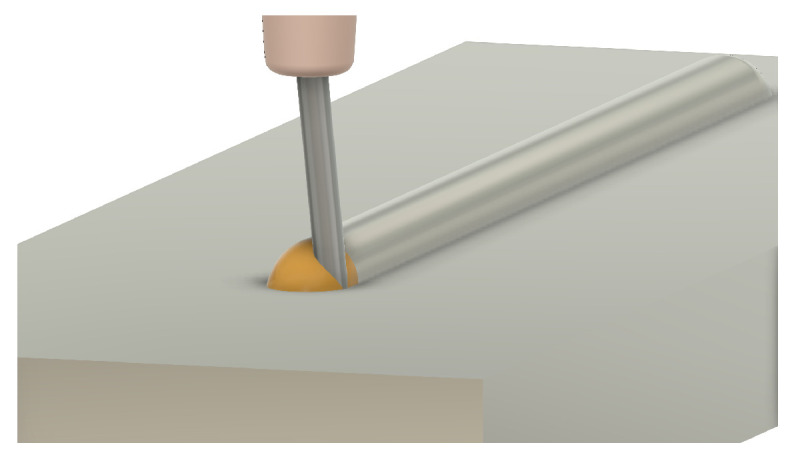
Three-dimensional rendering of a deposition that transitioned from stable to stubbing. The wire is no longer centered in the meltpool and is likely to begin creating ripples along the bead.

One of the first considerations when adjusting process parameters for a wire AM process is wire stability. Wire stability is a mechanism unique to wire-based processes that is a result of the physical connection between the deposition head and workpiece [4]. Wire stability is typically classified into three categories: dripping, stubbing, and stable [5]. The stability state of a deposition can be a result of process parameters such as laser power, feed rate, and wire feed speed [6,7]. The following is a description of each stability state:

**Stable**  —Stable deposition implies a smoothly moving, non-turbulent meltpool that enables the incoming wire to be fully melted. For a stable and coaxial process, the wire is centered in the meltpool during deposition. This stability state is typically preferred as it results in beads with a smooth surface finish and consistent geometry. A stable deposition is shown in Figure 1.
**Dripping**  —Dripping, also known as droplet formation, occurs when an excess of energy exists in a laser-wire system. The excess energy results in the overheating of the wire, which allows surface tension to pull the molten metal into a droplet. The droplet typically stays suspended above the workpiece until enough material has been fed for gravity to take over or until the droplet encapsulates the work tip, welding the system shut and resulting in a system failure. Dripping can also occur later in deposition due to excessive heat buildup in the workpiece [8]. A dripping deposition is shown in Figure 2.
**Stubbing**  —A lack of energy during deposition can result in stubbing. Stubbing occurs when the wire does not melt all the way, resulting in a collision with the bottom of the workpiece. In less serious circumstances, this contact causes the wire to wiggle in the meltpool, resulting in a poor surface finish for the bead. In more serious circumstances, the wire will stub outside of the meltpool, resulting in sections of unmelted wire. This can also result in the wire being welded to previous sections of the deposit, creating a system failure. A stubbing deposition is shown in Figure 3.

**Figure 4:**
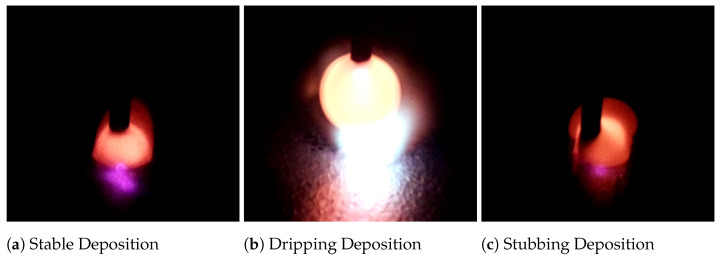
Deposition images captured with the RaspberryPi Cam Module 2. These images show stable, dripping, and stubbing deposits. During stable deposition, the wire remains in the center of the meltpool. During dripping deposition, the wire begins to wick up on itself, and an arc forms. During stubbing deposition, the wire moves from side to side within the meltpool.

Wire instability can have detrimental effects on both final part quality and machine integrity. Because of this, wire stability has been a research topic of interest for many years. Commonly, stability is characterized through the use of high-speed, high-dynamic-range, or normal video cameras [5,9,10]. Figure 4 shows a still image of each stability state captured by a standard visible light camera. In recent years, work has been carried out with these process monitoring cameras to train LSTM and CNN models as a means of defect detection [11,12]. In addition to defect detection, LSTM and CNN models have been used for meltpool anomaly and flaw detection [10,13]. This paper is novel in its implementation of a model-free classification and quantification technique for meltpool stability. By taking an engineered approach to the machine vision analysis, there is full transparency of function and flexibility of control. Every step of the algorithm is documented; there is no black box effect that is often associated with machine learning algorithms. This also gives the benefit of having a tuneable algorithm that can be adjusted to specific needs or situations.

## 2. Materials and Methods

### 2.1. Experimental Setup

**Figure 5:**
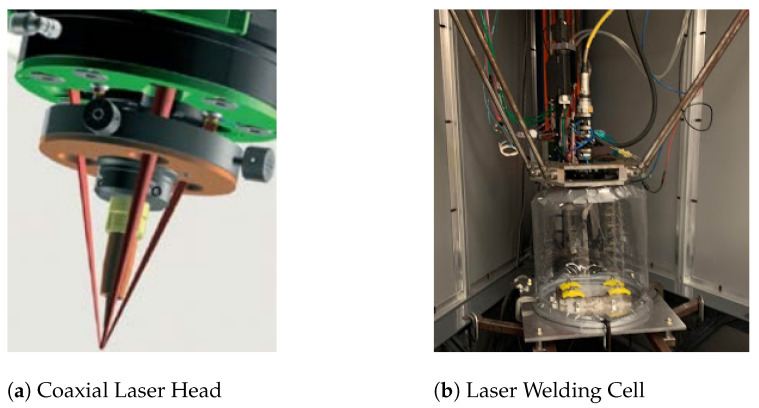
Setup of processing optic integrated with delta robot, laser input, and wire input [14]. (**a**) Fraunhofer COAXwire laser-wire Processing Optic. The single-input laser beam is split into three that distribute energy input around the wire which is fed perpendicular to the substrate [15]. (**b**) The processing optic is connected to the carriage of the delta robot. By controlling the actuation of the three vertical axes, coordinated motion in three orthogonal axes can be achieved.

All testing for this experiment was carried out on a laser-wire deposition cell fabricated by students at Missouri University of Science and Technology. Motion in the cell is facilitated through the use of a custom delta-style robot and controller. Bulk wire feed for the cell is provided by a Miller Auto-Continuum 500 (Miller, Milwuakee, WI, USA) and precision wire feed is provided by a Dinse DIX FD 200 M (Dinse, Wood Dale, IL, USA). Wire being fed through the system is 1.2 mm diameter Ti-6Al-4V and is deposited onto 6mm Ti-6Al-4V plates. The laser used for deposition is a near-infrared 4 kW Laserline LDF 4000-30 (Laserline, Livonia, MI, USA). The laser is passed into a Fraunhofer COAXwire (coaxworks, Dresden, Germany) laser-wire Processing Optic which splits the incoming beam into three separate beams that surround the central wire. Figure 5a shows the three beam laser-wire configuration. A small amount of hot-wire is used for additional heat input into the process and is provided by the Miller Auto-Continuum 500. In order to prevent oxidation of the deposited titanium structures, the environment is flooded with argon that is contained by a custom PVC welding tent. The base of the structure is connected to an Avani Enviromental Fume extractor (Avani Enviromental, Raleigh, NC, USA) that generates downward flow through the tent, keeping fumes away from the optics head. A picture of the fully assembled laser-wire cell is seen below in Figure 5b [14]. Research on the cell is supported by GKN Aerospace, who contributed the equipment, materials, and technical support.

**Figure 6:**
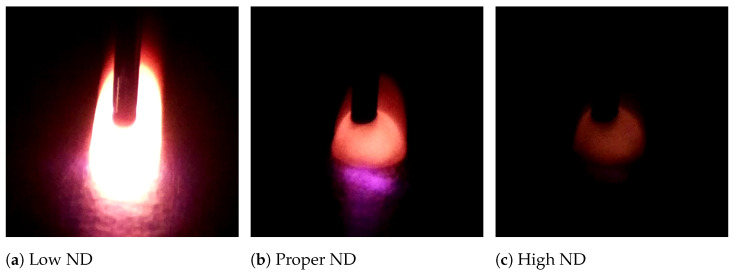
Varying levels of ND with same process parameters. The low-ND frame is very overexposed and therefore does not give very much information about the meltpool. Conversely, the high-ND frame blocks too much light out, once again hiding important features of the meltpool.

The camera used for this system is a Raspberry Pi Module 2 (Raspberry Pi Foundation, Cambridge, Great Britain). It records at 25 frames per second with 1080p resolution. The camera is mounted onto the delta robot’s frame and is focused at a distance of 0.25” from the wire nozzle. This ensures that the meltpool and bead remain in view and in focus at all times during deposition. The optics stack for the camera consists of a 16mm focusing optic, a near-IR hot mirror, and neutral-density (ND) filters. Hot mirrors are designed to reflect specific wavelengths of light and transmit others. Neutral-density filters are designed to block a portion of incoming light across all wavelengths. The hot mirror reflects all light above 720 nm and is used to eliminate laser effects from the image. The ND filters are used to reduce the overall brightness of the image. The amount of ND on the optic stack was tuned to provide an optimal deposition image. The ideal deposition image had as little ND as possible, while not allowing pure white pixels to exist during stable deposition. This setup preserves important details of the meltpool such as shape and intensity, while removing most of the harsh brightness from the laser. Because this algorithm is tracking visible light, for which emissivity variation is relatively low, the image processing technique should be effective for a variety of materials. Figure 6 shows various steps of the tuning process, with a range of ND spanning from too little to too much light attenuation.

### 2.2. Development of Machine Vision Tool

The machine vision tool developed in this study has two primary components: real-time analysis and post-deposition analysis. The real-time analysis portion of the tool utilizes a rolling buffer to analyze a small history of the deposition. This small history helps the tool decide when stubbing and dripping events have occurred and uses these events to classify what is currently happening in the deposit. The post-processing analysis portion functions similarly to the real-time analysis, but it considers the entire deposition history at once.

#### 2.2.1. Basic Tool Functionality

The primary driver of this tool is a dual-state machine. The two state machines keep track of the “deposition state” and “stability state”. The deposition state refers to the stage of the deposit that is currently occurring. Laying down a typical bead on this laser-wire cell will have four steps: trim, wire engage, deposition, and wire retract. Keeping track of the deposition state saves computational expense, as the stability state tool will only run during deposition. The second state machine, stability state, works to classify what is currently happening within the deposition. The three potential stability states are stable, stubbing, and dripping. The active stability state is recorded for every frame and is used at the end of testing to calculate deposition time statistics. The deposition state machine is necessary for this iteration of the machine vision tool because it is not yet integrated with the wire cell’s control system. Future work on the tool will include full machine integration, which will render use of the deposition state machine irrelevant, as the deposition state can be passed on from the machine controller.

**Figure 7:**
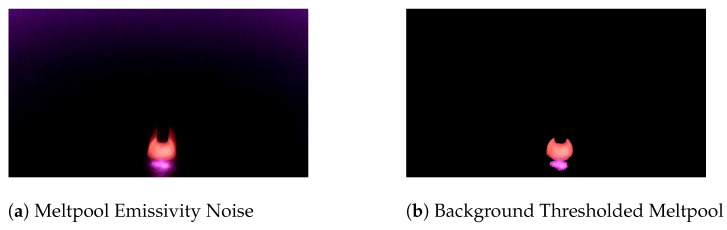
Stable deposition image before and after the background threshold is applied. A Gaussian blur is applied to the image before thresholding to reduce noise intensity. (**a**) Raw stable meltpool frame with radiation emission noise shown around the edges of the image. (**b**) Stable meltpool frame that has had noise removed from the initial background threshold.

**Figure 8:**
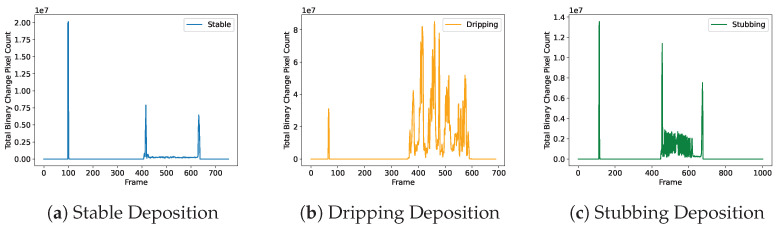
The binary change for various deposition states. This binary change value is the total pixel difference between back-to-back frames, which are thresholded into binary images. Stubbing deposition in (**c**) demonstrates clear spikes during events. Stable deposition in (**a**) is low throughout, while dripping deposition in (**b**) is an extremely strong and noisy signal.

This tool’s main mechanism for determining melt pool stability is through a “binary change” technique. The technique works by setting a threshold for every frame and converting it into a binary image. The threshold point for the frame is selected to remove the full image background while preserving the melt pool. Noise generated from radiation emission is eliminated through this initial background thresholding. A Gaussian blur is applied to the image, which reduces the intensity of emission noise. The reduced intensity causes it to drop below the background threshold and be extracted from the image. An example of this is shown in Figure 7. The created binary image is then laid on top of the previous frame’s binary image. The difference between them is calculated, leaving a map of 1s and 0s. In this map, 1s represent regions of the frame that have changed since the last frame, and 0s represent regions of that frame that remained the same. The sum of all remaining pixels is then calculated and stored for analysis throughout deposition. Figure 8 shows a plot of this “binary change” for stable, dripping, and oscillating deposits. The threshold used for this calculation is set statically. The value was determined empirically by recording stable bead depositions and assessing their effect on the binary change data. A pixel intensity value of 125 was used as the background threshold for all tests.

**Figure 9:**
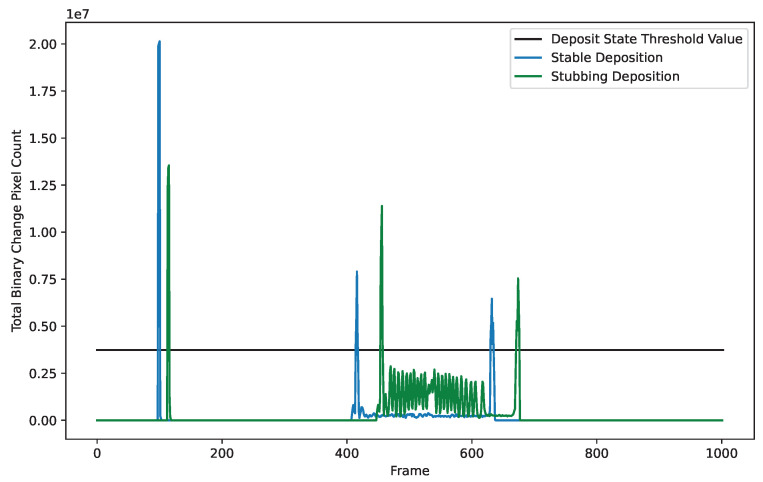
Deposit state threshold shown with stubbing and stable deposition. This threshold is used to signal that different positions of the deposit have started or ended. The first large spike shown is the wire trim, the second spike is initial engagement, and the final spike is the retraction.

The stable and oscillating plots shown in Figure 8a,c demonstrate a clear pattern that is consistent with what is physically happening during the deposition. The first spike in the data represents the wire trim before deposition starts. The second spike represents the initial engagement of the wire with the substrate, and the final spike in the data represents the wire’s retraction at the end of deposition. Because of the consistency and repeatability of these events within deposits, binary change is used as the sole driver for the deposit state machine. A threshold value is set that can capture each deposit state change, without being triggered by stubbing events. Figure 9 shows the deposit state change threshold for a variety of deposition states. Because dripping events are so much more turbulent than stable deposition or stubbing events, binary change is not used to detect the end of a deposit during dripping. Instead, the tool waits for a series of consistently blank images to know the deposit has finished.

#### 2.2.2. Detection of Deposition Events

**Figure 10:**
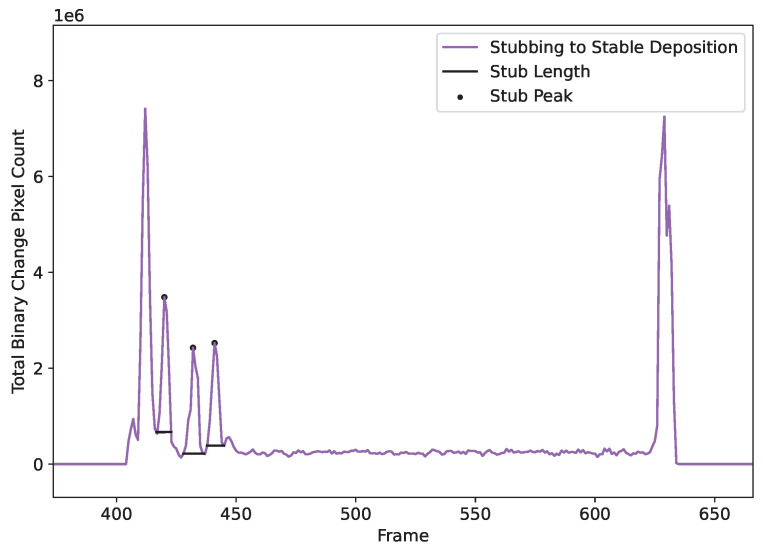
Binary change data showing stub detection as well as stub length calculation. The binary change pixel count is very responsive to movement within the meltpool.

Stubbing events are detected using the binary frame change data. Peaks in this data represent each event. The SciPy function find_peaks is implemented to detect data events [16]. This function takes a 1D array and finds all instances of local minima through a comparison of neighboring values. These minima can then be filtered and tuned so that only similarly relevant peaks are selected. In order to find the length of each peak, the first derivative of the 1D change array is calculated. Zero crossings nearest to events are then calculated and used as peak bases. Figure 10 shows an example of a stubbing deposit with peaks and peak lengths detected.

During real-time deposition, a rolling buffer is used to store binary change data for event detection. A rolling buffer is a first-in, first-out, fixed-length data structure used to transfer data from a producer service to a consumer service [17]. This utility is useful because it provides a means of regulating workflow between producer and consumer activities without needing to know the context of what is happening around it. In every frame, the peak detection function evaluates all data in the rolling buffer. If a peak is detected, the global index that correlates to the event is recorded. Use of the peak’s global index ensures that repetitions of the peak are not recorded as it moves through the rolling buffer. Because stub events happen quickly, it is important to maintain a history of past events to help influence current actions. Consider a rapid chain of three stubs happening within 1–2 s of each other. It makes more sense to classify the 1–2 s period as stubbing throughout, rather than to consider it as three discrete stubbing events woven with brief periods of stability. In order to account for this, the first stub in a series is given a configurable timeout length. If another event is not detected by the end of the timeout length, the series is considered over. If a stub event is detected within the initial timeout length, a new timeout length equal to the period between stub events is set. This ensures that as long as consistent oscillation is happening, the stability state machine is not repeatedly switching between stable and stubbing.

**Figure 11:**
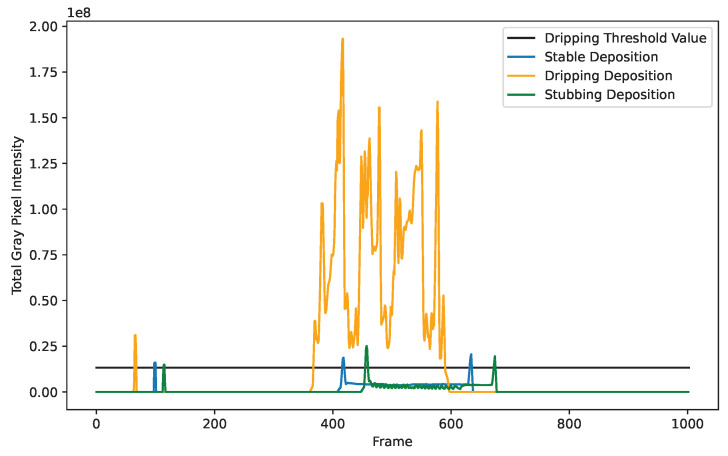
Dripping threshold shown with various deposition states. Because dripping events are so bright, they respond strongly to a cumulative sum of total pixel intensity.

Because of the intense and somewhat turbulent nature of dripping events, two different factors are used to influence their state selection. The first contribution to determine if the deposition is dripping is the intensity of the frame. Dripping events result in a large amount of energy being focused into a small volume of material. This high energy concentration results in a bright ball that looks white. This phenomenon is possible to see when looking at the cumulative sum of grayscale pixel values for each frame. To increase the robustness of this measurement, a mask is used to convert all pixels below the background threshold to 0 while preserving the grayscale value of all pixels above the background threshold. Using this technique, it is possible to set dripping and not dripping threshold valuses based on the total pixel intensity. Figure 11 shows a plot of this total intensity for stable, stubbing, and dripping deposits. To handle the turbulence of dripping events, a rolling buffer of frame intensities is also considered. The rolling buffer is averaged every frame, which accounts for any extremely bright or dull frames.

**Figure 12:**
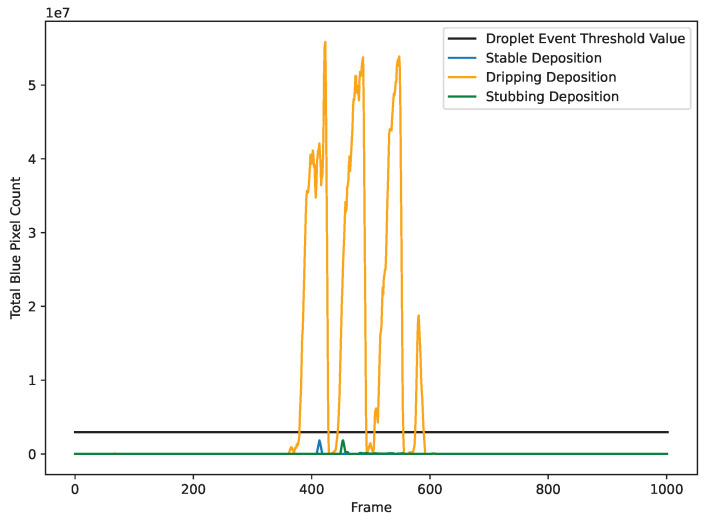
Droplet threshold shown with various deposition states. Because arcing only occurs during droplet formation, the amount of blue light in an image responds strongly during the phenomenon.

Although pixel intensity is a strong indicator of a deposit actively dripping, it does a poor job of identifying discrete drip formation. A method that can be used to help discretize this event is a frame color search. The reason a color search works is because of what is physically happening during this portion of the deposit—whenever a drip occurs, contact between the wire tip and substrate is lost, causing arcing to occur between the now elevated wire tip and the grounded plate below. Arcing releases a broad spectrum of light that is not typically seen throughout the deposit. By selecting a color of light that is not normally known to appear during deposition, it is possible to isolate the length and location of droplet formations. Figure 12 shows a plot of the total blue pixel quantity for dripping, stubbing, and stable deposits. Because overall intensity typically stays high between dripping events, and dripping events last much longer than stubbing events, it is not necessary to maintain a history of them to provide real-time feedback as to whether or not they are happening.

#### 2.2.3. Summary of Tool Design

The steps the machine vision tool takes every frame to calculate deposition stability are as follows:

1. Image Analysis
  (a) Pull the image from either a live camera view or a previously recorded video using OpenCV.
  (b) Create a grayscale image of the original frame.
  (c) Threshold the grayscale image to remove the background. Preserve all pixel values above the threshold and calculate a cumulative sum of all pixels. This value is saved as “Frame Intensity”.
  (d) Threshold the rest of the frame into a binary image. Add this frame into a rolling buffer.
  (e) Calculate the difference in the current binary frame and the previously saved binary frame from the ring buffer. Perform a cumulative sum of the difference, and save this value as the “Binary Change” count. Save the binary change value to a rolling buffer.
2. Deposition State Thresholding
  (a) Check the current state of the deposition state machine. There are six possible states that iterate in order: “Initialize”, “Trim”, “Engage”, “Deposition”, “Retract”, and “Complete”.
  (b) Monitor the “Binary Change” value to move between deposition states. This is triggered by the “Deposit State” threshold.
3. Stability State Analysis
  (a) Check the “Total Intensity” value. If this value is above the “Dripping Threshold”, then the stability state is “Dripping”.
    - If the system is dripping, pull blue pixel values from the original frame. Threshold these values to remove the previously set background pixels and pixels of maximum intensity. Save a cumulative sum of the remaining pixels as “Blue Pixel Intensity”.
    - Monitor the Blue Pixel Intensity throughout time spent above the “Dripping Threshold”. If this value rises above the “Droplet Event” threshold, record the location of drip formation.
  (b) If the system is not dripping, analyze the Binary Change rolling buffer for “peaks”.
    - If a peak is detected, consider it the start of a stubbing event.
    - Consider the stability state as stubbing until an “Initial Stub” wait time has passed.
    - If another event occurs within the “Initial Stub” wait time, calculate the period between stubbing events. This period will be used as the new wait time to see if another event will occur. If another event does not occur during this wait time, the stability state returns to stable.
  (c) If neither dripping or stubbing are observed, then the frame of the deposit is considered stable.

### 2.3. Experimental Design

In order to evaluate the feasibility and capability of the machine vision tool, a 1D parameter exploration experiment was designed. Using a simple 1D process window generated a wide range of stability states that allowed for comprehensive evaluation of the tool’s performance across all possible testing conditions. This is a 9-level linear space mapping experiment with 3 replications for each level. The laser power started at 1100 W, which was a known low-power instability state. The power was then increased by 50 W until a high-power instability state was reached, occurring at 1500 W. Replications were then performed for each parameter set. Every trial deposited was a 3-inch-long bead. Prior to the experiment, it was determined that 90 s was enough time for the substrate to return to a steady state. Therefore, a 90 s dwell time was included between each trial run to ensure ambient temperatures were equivalent for all tests. Prior to each test, argon was bled into the tent for 30 s to evacuate any oxygen from the environment. The camera was manually enabled before the wire trim of each test and manually disabled after retraction had completed for each test. After each test, data were post-processed using the machine vision tool detailed above, and the total time spent unstable was recorded.

#### Development of Bead Measurement Script

Bead analysis for this study focused on the external geometry impact of instability. This was deemed to be an effective method for analyzing stability effects on a single track bead. Gas porosity defects were not taken into account in this study but could be another defect mechanism created by instability. In order to evaluate bead profile effects from instability, a geometry analysis script was created. Beads were scanned using a Revopoint MINI 3D (Revopoint 3D, Shenzen, China) scanner with a precision of 0.02 mm. Prior to scanning, the beads were sprayed with AESUB Orange Scanning Spray (AESUB, Recklinghausen, Germany). This spray reduces reflections and improves scan quality. The spray has a layer thickness of 2–6 microns, which falls within the noise range of the 3D scanner. Point clouds were created from the scan data for each plate of beads. Each individual bead was then extracted from the plate and converted into an .STL file using RevoScan 5. Individual beads could then be passed into Python 3.11.5 for post-processing.

**Figure 13:**
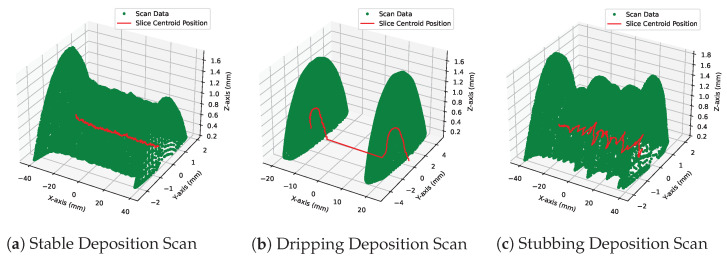
Three-dimensional scans for various qualities of beads. Green points are made up of 0.1 mm spaced slices along the bead. The red line passing through the beads is the centroid of each slice.

**Figure 14:**
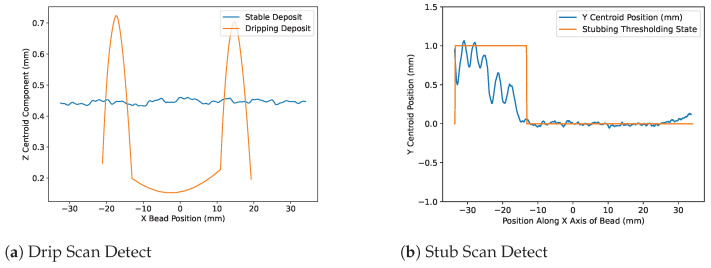
Plots from the scan detection script. (**a**) shows Z-centroid values plotted across a stable and dripping bead. (**b**) shows Y-centroid values for a stubbing bead and the threshold response from the data.

The goal of this measurement script was to provide a “Go, No-Go” means of assessing if a bead’s geometry could be considered acceptably stable. The “Go, No-Go” data label returned by the tool has 3 levels: Pass-Stable, Fail-Dripping, and Fail-Stubbing. The tool performs its analysis using the following method:

1. Geometry Import and Alignment—Beads are input into the software as .STL files. Principal Component Analysis is performed to find the X-Y-Z axes for the deposit. The deposit is then aligned to the X-Y-Z axes.
2. Slicing of Beads and Calculation of Centroid—Beads are sliced along the X axis in increments of 0.1 mm. The centroid of each slice is calculated and plotted through the center of the bead. Figure 13 shows a plot for beads of stable, Figure 13a; dripping, Figure 13b; and stubbing, Figure 13c, states, with the centroid line plotted through the center.
3. Threshold of Height for Drip Detection—Bead height is used to detect dripping because it is a robust scan measurement that has minimal effect from meltpool motion and is sensitive to rapid changes in material influx. This thresholding mechanism tracks the Z-component of the centroid line and signals a bad deposit if a specified height is reached. Figure 14a shows an example of Z-heights plotted for stable, stubbing, and dripping depositions.
4. Convolution of Y-Position for Stub Detection—A kernel convolution along with Y-centroid data is used to help mitigate the effects of noise from scan data. The Y-data are first thresholded by the bead width to find outlying points along the axis. The convolution then passes a kernel of 1s with width corresponding to the average period of stub events along the thresholded line. The resultant axis is then thresholded once more to determine the effect of stubbing events. Figure 14b shows an example of a stubbing deposit detected by this method.

## 3. Results

### 3.1. Output of Machine Vision Tool

**Figure 15:**
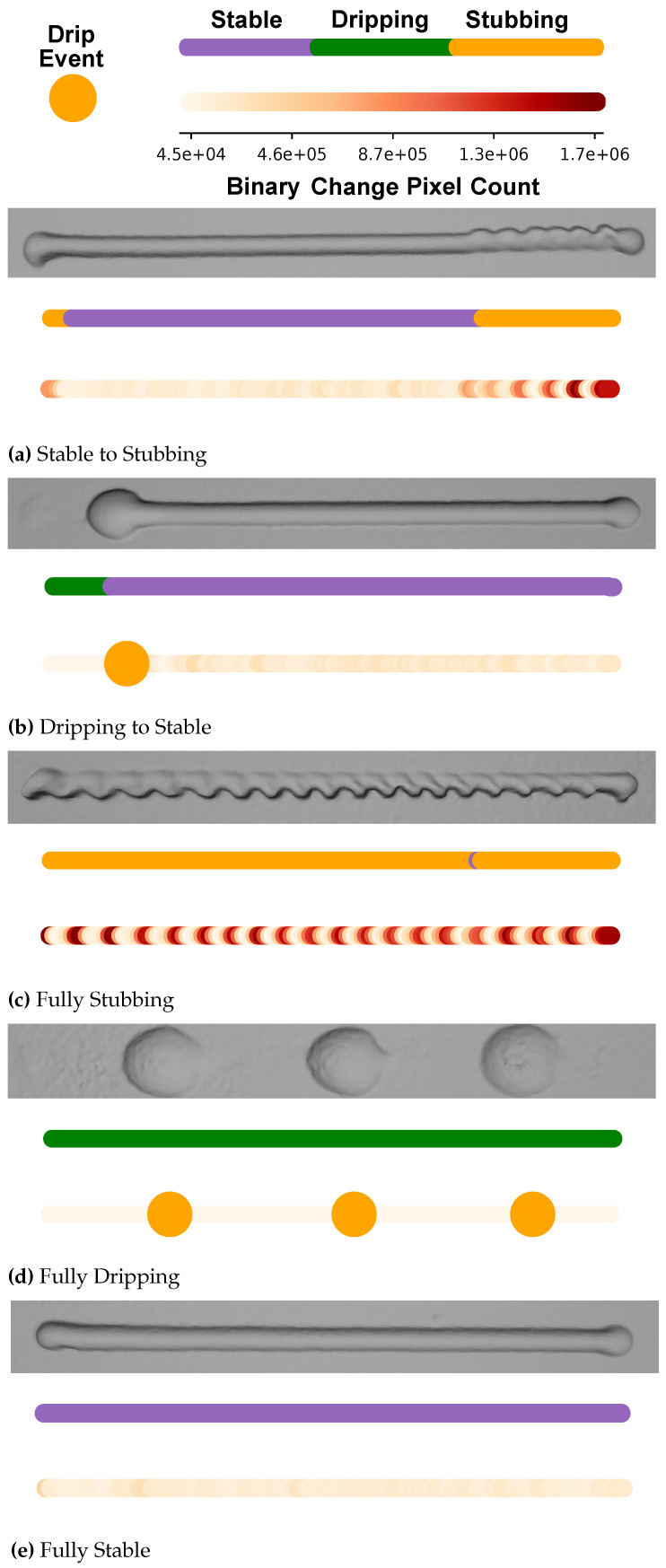
Machine vision tool results. Shown is a 3D scan taken of each bead, along with a data label map and heat map created by the machine vision tool. The data label map is colored for each state, and shows each frame along the deposition. The heat map represents the binary change calculated for each frame. A dot is placed along the heat map to show the location calculated for each drip event.

To validate the machine vision tool’s stability characterization, 3D scans were taken from beads that demonstrated a variety of stability states during deposition. These scans were then compared to a data label map created via machine vision analysis. To create the map, each possible stability state was associated with a color. Stable was purple, stubbing was orange, and dripping was green. At the end of the test, the stability state for each frame was plotted as a single dot the same color as its data label. The row of colorful dots creates a bead-like geometry that helps visualize classifications made by the algorithm. The stability states shown in each plot are stable to stubbing in Figure 15a, dripping to stable in Figure 15b, fully stable in Figure 15e, fully stubbing in Figure 15c, and fully dripping in Figure 15d. Stability state is only captured during deposition, which excludes engagement and retraction. For this reason, rendered beads are slightly shorter than scanned beads. In addition to the data label color map, a heat map was also created for each bead. This heat map plots the binary change recorded for each deposition frame as a colored dot. Layering these colorful dots once again creates a bead-like geometry, highlighting events noticed during deposition. In addition to plotting binary change for each bead, the location of dripping events is also plotted in the map. Whenever the deposit is classified as dripping, the binary change is set to 0, mimicking the bead being lifted off the plate.

### 3.2. Experimental Results

Results for the one-dimensional laser power vs. stability experiment are shown in Table 1. The experiment tested the amount of time each deposition spent unstable at varying laser powers. Unstable time is presented as a percentage of total deposition time. Negative values were assigned to unstable times that were designated as dripping. Table 1 shows measurement output from the previously mentioned bead scanning script. The output of the script is a series of data labels, identifying the deposit as containing stubs, containing drips, or being fully stable.

## 4. Discussion

### 4.1. Machine Vision Tool Results Discussion

The results shown in Figure 15a–e demonstrate a strong correlation between deposited beads and data obtained from the vision tool. Generally, the tool’s data labeling feature is able to track instability events continuously throughout a deposit. An exception to this is shown in the bead from Figure 15c. This bead has a quick stability change take place in between two large stub events. Rapid state changes like this could be problematic for use in a feedback control system. Mitigation of these rapid state changes comes with a trade-off of slower response time. The more time the tool takes to return to a stable state after an event, the higher the risk of it misinterpreting the current state as unstable. Another mistake that can be made by the data labeling system is shown in Figure 15a. Interpreting the transition from engagement to deposition as a stub event happened occasionally and was more likely to occur whenever the deposit engagement move did not start centered in a bead. This phenomenon represents another trade-off between measurement speed and sensor accuracy. Adding a longer pause for the sensor to begin collecting measurements will reduce these errors, but could result in missed early event detection. In order to increase measurement robustness and speed, it may be beneficial to incorporate additional deposition data with the tool. The use of active parameters such as wire feed speed or feed rate could be used to inform the tool of better oscillation event dwell times. Additionally, active deposition data such as meltpool brightness could help inform the tool of the beginning or end of a deposition event. One future research goal for this tool is to utilize it in a closed-loop control feedback system. The tool could have two potential real-time use cases. Firstly, if the tool is installed in a manufacturing environment where quality standards are less critical, it could be used to drive a process out of instability with the goal of preserving a deposited part. Secondly, the tool could be used as a machine safety feature. Dripping and stubbing can both easily damage a laser welding cell, so detecting these events happening and stopping them quickly would be beneficial.

**Figure 16:**
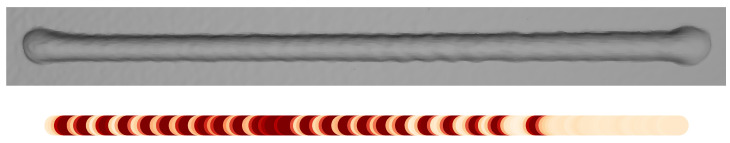
Deposition with less intense stubbing.

An observation made throughout experimentation with the tool is its inability to recognize stub severity within a bead. This effect can be seen when comparing the results in Figure 16 to the results in Figure 15c. During deposition, the bead in Figure 16 stubbed throughout the full process. However, oscillations during this event were centered about the middle of the bead. Because of this, the effect on the final bead geometry was reduced. Conversely, oscillations during deposition in Figure 15c were centered around the top edge of the meltpool, resulting in a much more dramatic effect on final bead shape. This is a problem because greater deviations in bead profile are more likely to result in inner bead defects, such as lack of fusion. The inability to quantify locational effects of stubbing within the meltpool makes sense, as the primary driver behind this tool solely relies on the rate of change within an image. This effect could be seen as having both favorable and unfavorable implications. The advantage is the ability to detect events irrespective of their physical size. This could aid in defect detection, where very slight changes in meltpool consistency induce flaws in a deposited part. The disadvantage is that the assignment of a “flaw intensity level” cannot currently be made. Another future research goal for this tool is to use it as a defect detection sensor during deposition. By recording when events are detected throughout a deposit, it may be possible to predict flaw locations. The efficacy of this tool would likely be greatly improved by the influence of meltpool position and geometry in its calculations. By implementing a tracking algorithm to complement the tool, it may be possible to predict resultant flaw size between beads in a deposition. Or more simply, it may be possible to predict the amplitude of profile deviations within beads. Although this paper focused on 1D single-bead deposits, a worthwhile application for the tool is to use it for tracking larger, multi directional deposits. In order to accomplish this, a second camera could be added orthogonal to the axis of the first view. Cameras in both directions will allow for superposition of calculations where travel directions obscure the field of view.

### 4.2. Experimental Results Discussion

**Figure 17:**
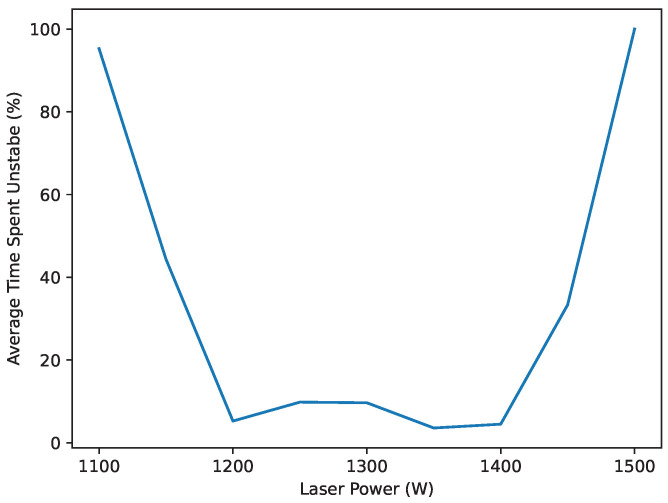
Stability experiment results. This plot shows the average time spent unstable for each laser power.

The results in Table 1 show a clear trend from more likely to stub at lower laser powers to more likely to drip at higher laser powers. Both ends of the parameter space demonstrated nearly full instability throughout the duration of their deposition for all replicates. Laser powers directly inside of the unstable parameter set, 1450 W and 1150 W, exhibit relatively chaotic behavior, made apparent by the high standard deviation between datasets for these powers. This behavior is likely a result of inconsistent start conditions from the process. Better parameter sets can overcome poor/inconsistent start conditions and drive the process towards stability. However, poor parameter sets are less likely to drive the process towards stability, and more likely to follow the behavior of their start condition. Laser powers between 1200 and 1400 W demonstrated the ability to drive the process towards stability. Figure 17 shows a plot of average instability time across the parameter window.

The bead scanning script was used to verify the results from the machine vision tool. Table 1 shows resultant data labels calculated for each experimental bead. Each data label is represented as a color within the cell it was calculated. These data perfectly align with data calculated by the machine vision tool. Only five stable beads were detected out of the 27 experimental runs. Sixteen beads were calculated to demonstrate some level of stubbing, and five beads were calculated to demonstrate some level of dripping. During experimentation, an experienced machine operator took notes based on observations from the live deposition camera. The operator classified each bead as stubbing, dripping, or stable at the end of deposit. The operator’s observations are recorded in Table 2. Highlighted in red are events that disagree with calculations from the machine vision and bead scanning tools. The operator properly classified 24 of the 27 tests, but mislabeled 3 unstable beads as stable. This test shows the propensity of human operators to make mistakes, especially when making decisions based off of fast-moving data.

## 5. Conclusions

The goal of this paper was to provide explanation and justification for an engineered machine vision algorithm that could provide a quantitative analysis of meltpool stability. The tool makes use of binary image sums and RGB colorspace sums to classify meltpool stability and identify instability events during deposition. Validity of the tool was confirmed through a 1D laser power study that compared machine vision results to 3D scan results. The machine vision results perfectly matched the scan results but differed from the operator testing observations. Development of this tool also yielded several branches for future study, including the following:

- Exploration of increased robustness of the tool through position-based meltpool analysis and active deposit data integration.
- Exploration of the tool as an in situ feedback sensor for process stability control.
- Exploration of the tool as a defect detection method for larger three-dimensional builds.
- Integration of a second camera with the tool to allow for multidimensional vision capabilities.

## Figures and Tables

**Table 1:** Machine vision and bead scan results for linear laser power stability experiment.

Laser Power	1100	1150	1200	1250	1300	1350	1400	1450	1500
Replicate 1	99.34	14.18	10.07	23.81	15.49	0.00	−13.47	0.00	−100.0
Replicate 2	97.94	27.14	1.38	0.00	6.25	8.57	0.00	−100.0	−100.0
Replicate 3	88.51	91.78	4.31	7.28	8.78	2.16	0.00	0.00	−100.0
Average	95.26	44.36	5.25	9.82	9.67	3.57	−4.49	−33.33	−100.0
Std. Dev.	0	41.36	4.42	12.43	5.06	4.46	7.76	57.73	0

Values shown in the table are percent of total deposition time spent unstable. Positive values are indicative of time spent stubbing while negative values are indicative of time spent dripping. The color in each cell represents the data label assigned to the deposition as calculated by the bead scanning script.

**Table 2:** Experimental stability observation table.

Laser Power	1100	1150	1200	1250	1300	1350	1400	1450	1500
Replicate 1	Stub	Stub	Stub	Stub	Stub	Stable	Drip	Stable	Drip
Replicate 2	Stub	Stub	Stable	Stable	Stub	Stable	Stable	Drip	Drip
Replicate 3	Stub	Stub	Stub	Stub	Stub	Stable	Stable	Stable	Drip

Values highlighted in red represent a disagreement between the operator observation of the process and results calculated by the machine vision and bead scanning systems.

## Data Availability

The original contributions presented in the study are included in the article. Further inquiries can be directed to the corresponding author.
